# Toward a Dynamic Follow-Up Protocol in Adolescent Idiopathic Scoliosis: Six-Month Out-of-Brace Evaluation as the Key Predictor of Treatment Success

**DOI:** 10.3390/children13010010

**Published:** 2025-12-19

**Authors:** Samra Pjanić, Vanja Dimitrijević, Bojan Rašković, Borislav Obradović, Nikola Jevtić, Theodoros B. Grivas, Filip Golić, Goran Talić

**Affiliations:** 1 Institute for Physical Medicine, Rehabilitation and Orthopedic Surgery “Dr Miroslav Zotović”, 78000 Banja Luka, Bosnia and Herzegovina; filipgolic@yahoo.com (F.G.); kancelarija.direktora@ms.zotovicbl.org (G.T.); 2 Faculty of Sports and Physical Education, University of Novi Sad, 21000 Novi Sad, Serbia; dimitrijevicvanja@gmail.com (V.D.); boriscons@yahoo.com (B.O.); 3 Functionally Aware Motoric Activity (FAMA) Center, 21000 Novi Sad, Serbia; 4 Performance Zone, 21000 Novi Sad, Serbia; 5 Scolio Centar, 21000 Novi Sad, Serbia; njevticns@gmail.com; 6 Physiotherapeutic Scoliosis-Specific Exercises Association (PSSE), 040251 Bucharest, Romania; 7 Department of Orthopaedics & Traumatology, “Tzaneio” General Hospital of Piraeus, 185 36 Piraeus, Greece; tgri69@otenet.gr

**Keywords:** adolescent idiopathic scoliosis, conservative treatment, short-term outcome, long-term outcome

## Abstract

**Highlights:**

**What are the main findings?**

- The six-month out-of-brace radiographic evaluation is the most reliable predictor of long-term brace treatment success in adolescents with idiopathic scoliosis.
- Lumbar and single-curve patterns show significantly better and more stable correction compared to thoracic or multiple-curve deformities.

**What are the implications of the main findings?**

- Clinical follow-up in AIS should focus on dynamic, time-dependent assessment rather than relying solely on initial in-brace correction.
- The six-month out-of-brace spine radiograph should be considered as an integral part of the standard clinical protocol for brace treatment evaluation to ensure more accurate assessment of treatment outcomes.
- Patients with thoracic or complex curve patterns require intensified monitoring and individualized treatment protocols to optimize outcomes.

**Abstract:**

Background: Adolescent idiopathic scoliosis (AIS) is a three-dimensional spinal deformity that requires effective conservative management to prevent progression and reduce surgical risk. Although bracing is effective, predictors of long-term outcomes and the prognostic value of short-term results remain insufficiently defined. Objective: This study aimed to identify clinical and radiological determinants of final treatment success in AIS patients treated with the Chêneau modified brace (CMB), with special emphasis on short-term predictors. Methods: A prospective longitudinal cohort study was conducted at the Institute for Physical Medicine, Rehabilitation, and Orthopaedic Surgery “Dr. Miroslav Zotović”, Banja Luka (2018–2024). Fifty AIS patients (mean age 13.5 ± 1.4 years; 80% female) with Cobb angles between 20 and 45° and Risser sign 0–3 were analyzed. Cobb angles were measured at four timepoints: baseline, 4 weeks (in-brace), 6 months (out-of-brace), and final follow-up (48.3 ± 9.4 months). Statistical analyses included repeated-measures ANOVA, Pearson correlation, and multivariate regression. Results: The mean Cobb angle improved significantly from 28.7° ± 7.1° to 22.1° ± 10.5° (*p* < 0.001). The six-month out-of-brace Cobb angle was the strongest predictor of treatment success (OR = 0.726, *p* = 0.001), surpassing initial in-brace correction. Lumbar curves demonstrated the most stable correction (28.1° → 20.5°, *p* < 0.001), while thoracic curves showed partial loss of improvement (30.5° → 26.9°, *p* = 0.373). Single-curve and non-compensatory deformities achieved greater correction than complex or double curves (*p* = 0.014). Conclusions: The six-month out-of-brace radiograph represents a key prognostic milestone in AIS management. Incorporating this dynamic assessment into routine follow-up may enhance individualized treatment planning and long-term outcomes.

## 1. Introduction

Adolescent idiopathic scoliosis (AIS) is the most prevalent spinal deformity in adolescence, affecting 1–3% of the global population, with a female predominance (2:1 to 8:1) [1,2,3]. Characterized by a three-dimensional deviation of the spine exceeding 10° Cobb angle without identifiable etiology, AIS typically manifests during puberty (ages 10–18) [4,5]. Progressive curves may lead to cosmetic concerns, pain, reduced pulmonary function, and psychosocial distress, particularly when exceeding 30° [6,7]. Early detection through school screening programs and radiographic monitoring is critical, as curve progression correlates strongly with skeletal immaturity (Risser sign 0–2) and growth velocity [8,9].

Conservative management, including physiotherapeutic scoliosis-specific exercises (PSSE) and bracing is the gold standard for moderate AIS (25–40° Cobb) in growing patients [10]. The Chêneau modified brace, a rigid thoracolumbosacral orthosis, employs three-dimensional corrective forces to derotate the spine and halts progression [11,12]. Its design incorporates expansion chambers for rib cage derotation and lumbar lordosis preservation, achieving in-brace corrections of 40–60% [13,14]. It is based on the original Chêneau brace concept, which employs a 3D correction strategy through the application of asymmetrical pressure zones and expansion voids to guide spinal realignment. The CMB refines the original design to enhance comfort, patient compliance, and biomechanical efficiency. By actively involving the patient in self-correction, it may also help maintain neuromuscular engagement and mitigate muscle hypotrophy. It is custom-made for each patient using CAD/CAM technology or mold-based systems. It is typically worn full-time (18–23 h per day) in patients with a high risk of progression, in accordance with current SOSORT recommendations and evidence from the BrAIST study [10].

Studies have shown that the Chêneau and its derivatives are effective in halting curve progression and even achieving partial correction in growing adolescents with moderate scoliosis curves [15,16]. Furthermore, its design aligns with the SOSORT and SRS recommendations for evidence-based bracing protocols [10]. Complementary PSSE approaches like Schroth, SEAS (Scientific Exercises Approach to Scoliosis), and BSPTS (Barcelona Scoliosis Physical Therapy School) enhance postural control, muscle symmetry, and brace compliance [17,18,19]. Advantages of conservative treatment over surgery include avoidance of invasive risks (e.g., infection, implant failure), preserved spinal mobility, and lower costs [10]. Bracing success rates reach 72–92% when adherence exceeds 90%, whereas surgery is reserved for curves > 45–50° with documented progression [20,21]. Recent long-term studies demonstrate that successfully braced patients maintain correction into adulthood with minimal degenerative changes [22,23]. Despite CMB’s widespread use in Europe, high-quality evidence predicting treatment outcomes remains limited. Prior studies identified in-brace correction > 50%, Risser sign progression, and adherence > 90% as potential success markers, but consensus is lacking [24,25,26].

Unlike our previous studies [27,28,29,30,31] which focused solely on the impact of exercise in the treatment of AIS and demonstrated its positive therapeutic effects, the present study investigates the combined effect of bracing and exercises in AIS patients. While this combined approach is standard, the prognostic factors that determine long-term success remain insufficiently defined, complicating clinical decision-making during follow-up. This study aims to address this gap by assessing the prognostic value of both clinical and radiographic measures taken during the first year of treatment, with the ultimate goal of evaluating which of these available early measures is the most reliable predictor for long-term outcome and thus optimizing the conservative management follow-up protocol.

## 2. Materials and Methods

### 2.1. Study Design

This was a prospective observational cohort study with subsequent retrospective follow-up and included patients with AIS treated using CMB at the Institute for Physical Medicine, Rehabilitation, and Orthopaedic Surgery “Dr Miroslav Zotović” in Banja Luka, Bosnia and Herzegovina, from December 2018 to August 2024. The initial 6-month prospective dataset was collected as part of the first author’s doctoral research and was conducted under a previous ethical approval issued before the start of prospective data collection. During this period, written informed consent was obtained from parents or legal guardians for participation in the study, including the use of anonymized patient data and participation in the adherence monitoring process.

After the prospective phase, patients continued brace treatment as part of routine clinical care, and long-term outcomes were obtained retrospectively from the Institute’s medical database. Ethical approval for the retrospective analysis of pre-existing clinical data was obtained from the Institutional Ethics Committee (Approval No. 21-40-12358-2/24, dated 10 June 2025). To clarify the distinction, all short-term clinical and radiological parameters (baseline data, in-brace correction, adherence monitoring, and 6-month outcomes) were collected prospectively according to the study protocol. Long-term outcomes were extracted retrospectively after treatment completion. All patients who completed brace treatment had complete long-term datasets, as these assessments are part of our standardized clinical protocol. This minimized the risk of missing data or retrospective bias, with only dropouts lacking full follow-up information. Given the long follow-up period, attrition was anticipated and addressed through sensitivity analyses to ensure the robustness of our findings.

### 2.2. Study Population and Eligibility Criteria

All participants were diagnosed with AIS and met the established criteria for brace treatment. Eligible patients presented with a primary structural scoliotic curve measuring between 20° and 45° according to the Cobb method and demonstrated skeletal immaturity corresponding to Risser sign 0–3 based on the U.S. classification system. None of the participants had previously undergone brace treatment.

Patients were excluded if they were younger than 10 or older than 16 years, had secondary scoliosis associated with neuromuscular, congenital, or syndromic conditions, demonstrated a Risser sign of 4, or presented with a spinal curve measuring less than 20° or greater than 45° according to the Cobb method.

The indication for brace treatment was determined by the attending physicians following standard clinical procedures that included anamnesis, physical examination, and radiographic assessment. A full-spine standing anteroposterior (AP) radiograph served as the basis for treatment decision-making. All radiographs were obtained using pediatric radiation protection standards, including breast shielding for female patients and gonadal protection for both sexes. Radiographic criteria included a documented scoliotic curvature of ≥20° ± 5° with evidence of skeletal immaturity and an elevated risk of progression, commonly observed during the pubertal growth spurt. In addition to radiographic parameters, physicians also considered clinical factors such as trunk imbalance, aesthetic deformity, family history of scoliosis, and generalized joint laxity when determining treatment initiation.

### 2.3. Intervention

The Chêneau modified brace was prescribed and individually designed following the initial clinical and radiographic assessment. During the treatment period, patients were regularly monitored by a multidisciplinary team consisting of physicians specialized in Physical and Rehabilitation Medicine (PRM), orthotists, and physiotherapists trained in PSSE, all working in close collaboration. In addition to brace treatment, all patients participated in a structured physiotherapy program based on the Schroth method, implemented before, during, and after brace fabrication. The program was implemented to facilitate brace adaptation, promote corrective postural alignment, and support the continuous performance of therapeutic exercises during brace wear and throughout daily activities. Because all participants received both brace treatment and Schroth physiotherapy as an integrated standard-of-care approach, the individual contribution of each modality to the outcomes cannot be separated.

The physiotherapy schedule included a three-week period of daily supervised Schroth sessions during the fabrication of the first brace, followed by a prescribed daily home exercise program that patients were instructed to perform throughout the treatment period, with annual refresher sessions provided at our clinic. However, adherence to the home exercise program was not systematically monitored, and therefore the extent to which physiotherapy influenced treatment outcomes cannot be fully determined.

Brace wear duration was prescribed individually according to the assessed risk of curve progression. In this cohort, which consisted predominantly of high-risk patients, full-time brace wear (18–23 h per day) was recommended.

Treatment adherence was evaluated using a custom-designed compliance questionnaire developed by the multidisciplinary team. Brace-wear adherence in this cohort was monitored only during the initial six months and relied on subjective self-reporting of total wearing hours and general day–night patterns. Therefore, these data were not considered sufficiently robust or longitudinally comprehensive to support further compliance stratification in the present analysis.

### 2.4. Outcomes and Measurements

The primary radiographic outcomes were the Cobb angle measurements at baseline, at 6 months of treatment (short-term outcome), and at the end of the treatment protocol (long-term outcome). Secondary outcomes included in-brace correction and correction rates both in and out of the brace.

Treatment success was defined as a final Cobb angle < 30°, a threshold widely used in longitudinal brace outcome studies and consistent with SOSORT and SRS concepts of long-term curve stability. Curves remaining below this level at or near skeletal maturity have a minimal likelihood of adult progression and typically do not require surgical management [1,10,32].

Demographic and clinical parameters included age, sex, height, weight, and menarcheal status in girls, as well as scoliometer readings, i.e., measurements of the angle of trunk rotation (ATR) at the level of the primary curve, alongside clinical evaluation of coronal and sagittal balance. Radiographic parameters included the Cobb angle and apical vertebral rotation of the primary curve (measured using the Raimondi method), coronal balance, and Risser sign.

All measurements were performed by trained physicians experienced in scoliosis assessment, with four physicians contributing to these evaluations over the study period using standardized techniques. Although multiple assessors were involved, ATR, Cobb angle and apical vertebral rotation have demonstrated high intra- and inter-rater reliability in the literature when standard procedures are followed. The inter-observer variability of Cobb angle is particularly relevant; meta-analytic evidence indicates an inter-observer measurement error of approximately 3–5° [33,34]. In addition, the validity and reliability of the Raimondi method for assessing apical vertebral rotation have been confirmed in comparative radiographic studies, demonstrating strong agreement with established rotation-measurement techniques [35,36].

Cobb angle was measured at four time points during the study: initially (baseline), after 4 weeks, after 6 months, and at the end of treatment. At baseline, clinical examination and radiographic evaluation (anteroposterior and lateral projections) were performed, forming the basis for the prescription and fabrication of the Chêneau modified brace. Four weeks after brace application and adaptation to the prescribed wearing schedule, an in-brace AP radiograph was obtained to assess brace effectiveness. At 6 months, a follow-up AP radiograph was performed out of the brace, after a 24 h brace-free period, to evaluate short-term treatment effects. The final radiograph was obtained at the end of the treatment protocol, following gradual brace weaning, after the patient had not worn the brace for 5 consecutive days, in accordance with the Institute’s standardized procedure.

These follow-up time points correspond to established clinical milestones in AIS brace management: the 4-week in-brace radiograph is routinely used to verify corrective effect and guide brace adjustments, whereas the 6-month out-of-brace radiograph has been included as a potential early prognostic indicator of long-term treatment success.

A 24 h brace-free interval was applied at 6 months to minimize short-term rebound effects in patients who were braced full time at that stage of treatment.

A 5-day brace-free interval at the end of treatment reflected the final weaning stage, when patients were wearing the brace only at night, allowing a stable assessment of the definitive spinal alignment without risk of short-term post-removal collapse.

There is no standardized out-of-brace (OOB) protocol in the literature, with reported intervals ranging from 2 h to 7 days depending on the treatment phase and study aims [4,14]. However, our choice of two different OOB durations was phase-specific and clinically driven. The shorter 24 h interval reduced the risk of temporary post-removal collapse in full-time wearers, while the longer 5-day interval at treatment completion allowed the curve to equilibrate and reflect true structural alignment independent of brace effects.

While the use of different OOB intervals may introduce some measurement variability, the absence of standardized guidelines for OOB timing and the consistent application of each interval across all patients at the corresponding time point limited its potential impact on the interpretability of longitudinal outcomes.

All radiological assessments were performed using the same digital X-ray unit (PROTEUS XR, GE Healthcare, Chicago, IL, USA) with the Carestream Classic CR image processing system (Carestream Health, Rochester, NY, USA), maintaining standardized patient positioning for every examination. During treatment, brace-wearing hours were periodically adjusted according to radiological findings and growth status. The Institute’s weaning protocol followed the SOSORT guidelines, recommending gradual reduction in brace-wearing time once the following criteria were met: a stable Cobb angle, Risser sign 4, no measurable growth during the last 3 months, or less than 1 cm of growth during the last 6 months. The brace-wearing duration was then reduced by 2 h every 4 months until complete discontinuation.

### 2.5. Statistical Analysis

Statistical analysis was performed using SPSS Statistics for Windows, version 29.0 (IBM Corp., Armonk, NY, USA), while statistical power was estimated post hoc with G*Power (version 3.1.9.7, Universität Düsseldorf, Düsseldorf, Germany). The normality of data distribution was tested using the Shapiro–Wilk test. Quantitative continuous variables were expressed as mean values with standard deviations. Differences between groups were compared using independent *t*-tests, while changes in the Cobb angle across the four measurement time points were analyzed with repeated-measures ANOVA. Pairwise comparisons were conducted with Bonferroni correction for multiple testing.

Pearson’s correlation coefficient was applied to assess the strength of the association between radiographic parameters and the final Cobb angle. Differences between initial and final values of the angle of trunk rotation (ATR) were examined using a paired t-test. Predictors of treatment outcome were evaluated using both univariate and multivariate linear regression analyses, with the Cobb angle measured at the end of treatment as the dependent variable. Independent variables included the initial Cobb angle, Cobb angle after 4 weeks, Cobb angle after 6 months, which were treated as continuous measures expressed in degrees with an increment unit of 1°, as well as the initial and final ATR values. To determine the most parsimonious and statistically efficient subset of predictors, thereby mitigating the risk of model overfitting, a Stepwise linear regression analysis was also performed. Multicollinearity among predictors was assessed using the Variance Inflation Factor (VIF), with values above 10 considered indicative of severe collinearity. The discriminative ability of the Cobb angle measurements (initial, 4 weeks, and 6 months) to predict the long-term treatment outcome (defined by a clinical threshold of 30°) was assessed using ROC analysis, evaluating two scenarios: treatment failure (Cobb ≥ 30°) and treatment success (Cobb < 30°).

Missing long-term outcome data were initially addressed using complete case analysis. To test the robustness of our primary findings, we performed multiple imputation with 20 imputations using logistic regression models based on baseline and interim radiographic measures. Pooled estimates were subsequently derived according to Rubin’s rules.

Statistical power was estimated post hoc using pseudo R^2^ values derived from the logistic regression model (converted into f^2^). For the initial Cobb angle (R^2^ = 0.36), the four-week Cobb angle (R^2^ = 0.393), and the six-month Cobb angle (R^2^ = 0.485), the estimated power exceeded 0.90, with the highest value observed for the six-month measurement (>0.95). Although the sample size was reduced from the planned 80 to 50 patients due to treatment discontinuation, the statistical power of the key findings remained high, while in the full sample it would have approached its maximum (≈1.00).

## 3. Results

### 3.1. Patient Characteristics

**Figure 1:**
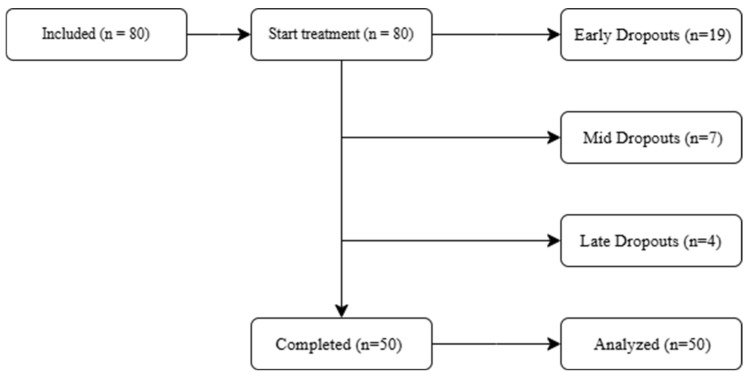
Flow diagram of the participants.

A total of 80 patients aged 10–16 years were included in the study and initiated brace treatment. Of these, 50 patients completed the full treatment protocol (mean duration 48.29 ± 9.36 months), whereas 30 discontinued treatments at various stages (Figure 1). The specific reasons for discontinuation could not be determined, as these patients stopped attending scheduled follow-up visits, and no further information was available in their clinical records.

Baseline demographic and clinical characteristics did not significantly differ between completers and dropouts in terms of age, sex distribution, curve location, initial Risser sign, or initial Cobb angle. This indicates that the analyzed cohort was representative of the full sample initially enrolled (Table 1).

The final analysis included 50 AIS patients, of whom 40 (80%) were female and 10 (20%) male, with a mean age of 13.5 ± 1.4 years at treatment initiation. As expected for a growing adolescent cohort, there was a significant increase in anthropometric parameters during follow-up: mean body height increased from 166.83 ± 9.45 cm at baseline to 172.96 ± 8.57 cm at the final evaluation, while mean body weight increased from 52.75 ± 12.35 kg to 61.52 ± 12.41 kg. These changes confirm that brace treatment was conducted predominantly during the period of accelerated growth, which is considered the phase of highest risk for scoliosis progression.

Skeletal maturity parameters followed the same pattern. The mean Risser sign increased from 1.47 ± 1.18 at baseline to 4.70 ± 0.39 at the end of treatment, indicating that most patients reached or approached skeletal maturity by the time the brace was discontinued. The mean total treatment duration was 48.29 ± 9.36 months (range 29.77–71.47), which reflects long-term brace management in accordance with the institutional protocol (Table 2).

Notably, this level of loss to follow-up is consistent with rates reported in other long-term AIS brace cohorts, where discontinuation of scheduled visits is common due to the prolonged duration of treatment [4,37,38,39].

### 3.2. Curve Type and Correction Outcomes

When patients were stratified according to curve pattern, clear differences in treatment response emerged. Patients with single-curve scoliosis achieved a mean correction of 11.4° ± 6.1°, which was almost twice the correction observed in patients with multiple-curve scoliosis (5.3° ± 7.2°, *p* = 0.014). This indicates that curve complexity is an important determinant of brace effectiveness and that simpler deformities respond more favorably to conservative management (Table 3).

A similar pattern was observed when the presence of compensatory curves was taken into account. Patients without compensatory curves had a mean correction of 11.3° ± 6.5° whereas those with compensatory curves achieved only 5.5° ± 7.2° (*p* = 0.023). Considering the established ±5° measurement error for the Cobb angle [40], and that changes within this threshold generally do not reach the minimal clinically important difference, the observed variability suggests that the distribution of values in both groups encompassed both clinically meaningful improvements and slight curve progression.

Compensatory curves often reflect a more three-dimensional, structurally fixed deformity. This finding suggests that their presence limits the potential for long-term radiological improvement, even when brace wear is adequate (Table 3).

### 3.3. Longitudinal Cobb Angle Analysis

Cobb angle was evaluated at four predefined time points (baseline, 4 weeks in-brace, 6 months out-of-brace, and final follow-up) in order to determine both the immediate mechanical effect of the brace and the sustained structural effect during growth. Repeated-measures ANOVA demonstrated a highly significant change in Cobb angle over time (*F* = 101.18, *p* < 0.001) with a very large effect size (η^2^ = 0.87), indicating that time, i.e., the course of brace treatment accounted for 87% of the variance in Cobb angle. This very high η^2^ value confirms that the observed changes were not random fluctuations, but rather a consistent treatment effect.

At baseline, the mean Cobb angle was 28.7° ± 7.1°. After 4 weeks of brace wear (in-brace radiograph), there was a marked mechanical correction with a mean reduction of 15.96° compared to baseline, reflecting effective force application and adequate brace fitting. However, the more clinically meaningful measurement was at 6 months out of the brace, when patients were instructed to remove the brace 24 h before imaging. At that time point, the mean improvement was 7.92° compared to baseline, which represents the true short-term effect of brace treatment independent of the immediate mechanical constraint.

At the final follow-up, after long-term brace wear and subsequent weaning according to the Institute’s protocol, the mean Cobb angle was 22.1° ± 10.5°, corresponding to a sustained correction of 6.64° (*p* < 0.001) relative to baseline. Since correction at 6 months and at final evaluation were very close (20.8° vs. 22.1°), this suggests that the majority of structural correction was achieved within the first 6 months and then maintained, rather than progressively increasing over the following years. This finding is an important clinical message, supporting the concept of early out-of-brace evaluation as a prognostic milestone (Table 4).

Angle of trunk rotation (ATR), as a clinical correlate of vertebral rotation and rib prominence, also improved significantly from 9.0° ± 3.5° at baseline to 5.2° ± 3.2° at the end of treatment (mean change 3.9° ± 3.9°, *p* < 0.001), indicating that the brace regimen had a measurable impact not only on coronal alignment but also on the rotational component of the deformity.

### 3.4. Regional Differences

Because brace behavior and corrective forces differ according to curve location, Cobb angle progression was additionally analyzed separately for thoracic, thoracolumbar, and lumbar curves. Repeated-measures ANOVA with Bonferroni correction showed statistically significant time effects for all curve locations, thoracic (*F* = 23.913, *p* < 0.001, η^2^ = 0.62), thoracolumbar (*F* = 13.715, *p* < 0.001, η^2^ = 0.48), and lumbar (*F* = 67.157, *p* < 0.001, η^2^ = 0.82) but the magnitude and durability of the correction differed (Table 5).

The lumbar curves (*n* = 28) showed the most favorable and the most stable response, improving from 28.11° to 20.54° at final follow-up (*p* < 0.001), with very small loss of correction between 6 months and the end of treatment (–0.86°, *p* = 1.000). This suggests that in this cohort, lumbar scoliosis was the most responsive to Chêneau-type bracing.

Thoracolumbar curves (*n* = 7) also improved significantly from 27.43° to 17.86° (*p* = 0.006), although a certain degree of correction loss after the 6-month time point was observed. This pattern implies that thoracolumbar curves may require longer full-time brace wear or slower weaning to preserve the achieved correction (Table 5).

In contrast, thoracic curves (*n* = 15), although they showed a large initial in-brace correction (30.47° → 16.67°, *p* < 0.001), did not maintain the same level of improvement in the long term. The final mean Cobb angle was 26.93°, which was not significantly different from baseline (30.47°, *p* = 0.373), indicating partial loss of the early effect. Clinically, this supports the conclusion drawn from the abstract that thoracic and more complex curve patterns require intensified monitoring, closer follow-up intervals, and possibly combined approaches (brace therapy plus specific physiotherapeutic exercises) (Table 5).

### 3.5. Predictors of Treatment Success

To identify which measurement time point best predicts long-term treatment success, correlation, logistic regression, and multiple linear regression analyses were performed. Pearson correlation analysis showed that all Cobb angle measurements across time were positively and significantly correlated, with the strongest relationships observed between the initial and six-month Cobb angles (r = 0.865) and between the six-month and final Cobb angles (r = 0.795). This pattern clearly indicates that the six-month out-of-brace measurement captures the clinically relevant treatment response more accurately than the immediate in-brace correction at four weeks (Table 6).

Logistic regression confirmed this finding. The six-month Cobb angle was the strongest independent predictor of achieving a favorable final outcome (defined as final Cobb < 30°), with an odds ratio of 0.726 (*p* = 0.001) and a pseudo-R^2^ of 0.485, indicating that nearly half of the variance in treatment success could be explained by the six-month result alone. Initial and four-week Cobb angles were also significant in univariate models (OR 0.759 and 0.784, respectively), but lost statistical significance in the multivariate model, likely due to shared variance with the six-month Cobb angle. This finding strongly supports the main hypothesis of the study, that early in-brace correction alone is not sufficient, and that an additional out-of-brace evaluation at six months provides essential prognostic information.

When improvement was defined more strictly (improvement > 5°), early in-brace correction (initial → 4 weeks) was not a significant predictor (*p* = 0.092), whereas the correction achieved by six months was (*p* = 0.009). Similarly, the percentage correction rate at six months showed a stronger association with final outcomes than the correction rate at four weeks. This indicates that the quality of maintained correction, rather than the initial mechanical effect, has greater prognostic value.

**Figure 2:**
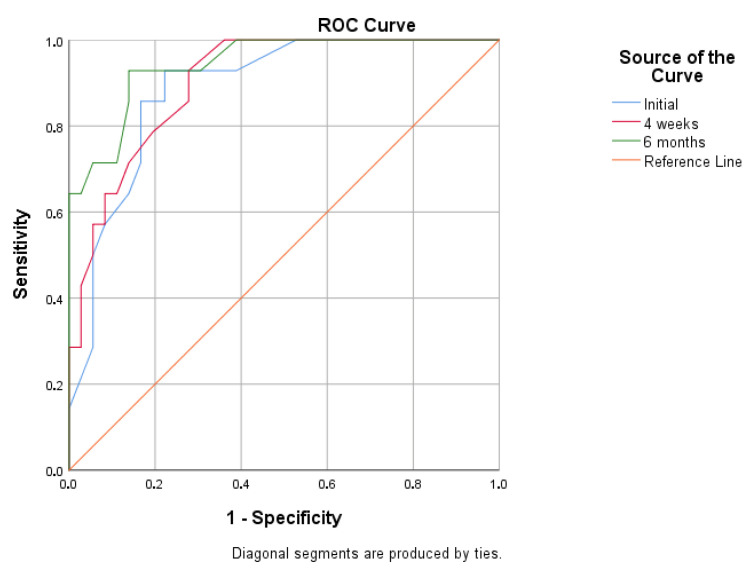
Receiver Operating Characteristic (ROC) curves for predicting treatment failure, defined as Cobb angle ≥ 30° (Positive Outcome).

**Figure 3:**
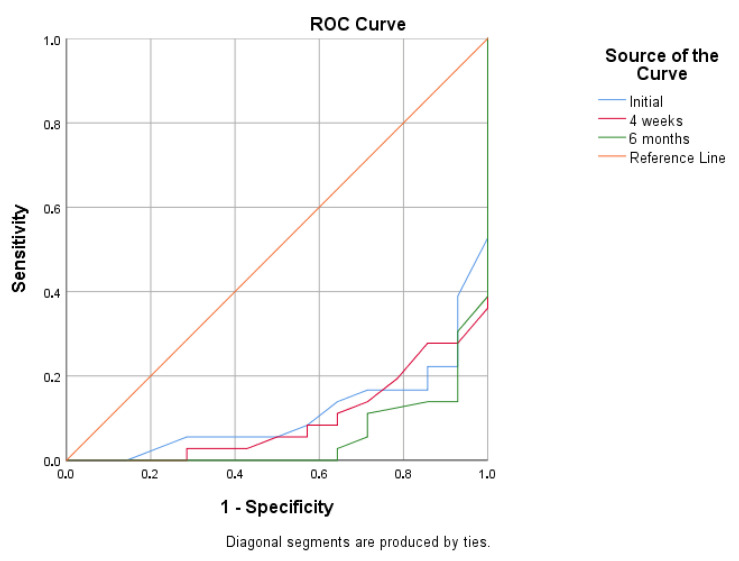
Receiver Operating Characteristic (ROC) curves for predicting treatment success, defined as Cobb angle < 30° (Positive Outcome).

ROC analysis demonstrated high AUC values, indicating a strong discriminative ability of all monitored angles in predicting treatment outcome (Table 7). The six-month out-of-brace measurement showed the highest predictive value (AUC = 0.944; 95% CI: 0.882, 1.000; *p* < 0.001), while the initial angle and the 4-week angle also exhibited high discriminative ability (AUC = 0.888 and 0.903, respectively). When treatment success was defined as a positive outcome, the ROC analysis yielded inverted AUC values (Figure 2 and Figure 3). Analysis of the ROC curve coordinates and the Youden index revealed that the majority of cut-off values yielded high sensitivity but very low specificity, resulting in negative Youden index values and preventing the statistical determination of a clear optimal threshold.

The multiple linear regression model, which included Cobb angles at baseline, four weeks, and six months, as well as initial and final ATR, explained 75.5% of the variance in the final Cobb angle (R^2^ = 0.755, adjusted R^2^ = 0.726, *F* = 26.446, *p* < 0.001), which represents a robust model for a clinical study with 50 participants. Residual diagnostics confirm the necessary statistical assumptions: the residuals follow an approximately normal distribution (P-P Plot and Histogram), and the scatterplot of residuals shows a random pattern around zero, supporting homoscedasticity and linearity. However, in the fully adjusted model, only the six-month Cobb angle (Adjusted B = 0.508, *p* = 0.004), final ATR (Adjusted B = 0.612, *p* = 0.004), and initial ATR (Adjusted B = –0.997, *p* < 0.001) remained significant independent predictors. All VIF values were below the threshold of 10, indicating no serious multicollinearity, although Cobb angle measurements showed moderate overlap (Table 6). To ensure the most parsimonious model and to address the risk of overfitting inherent to the *n* =50 sample size, a Stepwise linear regression was additionally performed. The Stepwise analysis resulted in a model with only one predictor: Cobb angle six months. This simpler model, which explains 63.3% of the variance (R^2^ = 0.633), was selected as the most robust and generalizable option, as the Stepwise algorithm determined that no other variable significantly added to the predictive power of the Cobb angle six months measure alone (all excluded predictors, including ATR, showed *p* > 0.05 for independent entry into the model). This supports the finding that the six-month Cobb angle is the dominant single factor in predicting final outcome.

### 3.6. Sensitivity Analysis

Sensitivity analysis using multiple imputation supported the primary results. While the six-month Cobb angle remained the strongest predictor of treatment success, initial and four-week Cobb angles also continued to show significant associations with outcome in the imputed models. The concordance between complete-case and imputed analyses indicates that the findings are robust despite attrition (Table 8).

## 4. Discussion

### 4.1. Summary of Results

This longitudinal cohort study aimed to identify key clinical and radiological predictors of long-term outcomes, as well as the relationship between short-term and long-term treatment outcome in braced patients with AIS. The results demonstrated a statistically significant improvement in spinal alignment, with the mean Cobb angle decreasing from 28.7° at baseline to 22.1° at final follow-up (*p* < 0.001). Special attention was given to ensure that the correction of the primary curve was achieved without inducing the development or progression of secondary (compensatory) curves. Patients with single-curve scoliosis and without compensatory curves achieved nearly double the correction compared to those with more complex curve patterns. The six-month Cobb angle emerged as the strongest independent predictor of treatment success, surpassing the prognostic value of initial in-brace correction. In addition, both initial and final ATR were independently associated with outcomes, highlighting the role of clinical rotational deformity in treatment response. Lumbar and thoracolumbar curves showed the most stable improvements, whereas thoracic curves demonstrated a partial regression of early gains.

### 4.2. Clinical Implications

For patients, these findings emphasize the critical importance of maintaining consistent brace wear during the early treatment phase, particularly within the first six months. We attribute the outcomes obtained to the substantial role of a multidisciplinary team in both the treatment and follow-up of our patients. This is consistent with previous research [31], which has demonstrated that a coordinated multidisciplinary team approach plays a crucial role in improving patient compliance and long-term engagement, ultimately leading to better treatment outcomes.

Achieving significant correction early in the treatment process was shown to be a strong indicator of successful long-term outcomes, reinforcing the value of patient motivation and support systems in sustaining adherence. For clinicians, this study supports the prioritization of the six-month radiographic evaluation as a pivotal checkpoint in treatment planning. The data suggest that mid-term outcomes are more indicative of final results than immediate in-brace correction, underlining the need for continuous monitoring and adaptive decision-making. These findings are directly corroborated by previous research [41], which has shown that the first out-of-brace radiograph provides a more accurate prediction of long-term treatment outcomes compared to both in-brace and baseline images. The six-month Cobb angle emerged as the strongest independent predictor of long-term treatment success, confirming its role as a robust clinical marker. This is supported by the Stepwise regression analysis, which identified the six-month Cobb angle as the sole independent predictor in the most parsimonious model, explaining 63.3% of the variance in final outcome and demonstrating superior predictive efficiency over other measurements. This robust finding advocates for prioritizing the six-month radiographic evaluation as the pivotal checkpoint in treatment planning.

The inclusion of both radiographic measures (Cobb angle) and clinical parameters (ATR) within the predictive model supports a comprehensive assessment strategy. Persistent rotational deformity at the end of treatment and the degree of initial trunk rotation are meaningful indicators of structural response and correction potential. Understanding the differential responsiveness of various curve patterns, particularly the challenges associated with thoracic curves, may help tailor more effective, individualized treatment strategies. Identifying curve type and compensatory mechanisms early on can inform the frequency of follow-ups and adjunct therapies, such as physiotherapeutic scoliosis-specific exercises.

### 4.3. Scientific Significance

This study contributes important evidence to the growing literature supporting conservative management of adolescent idiopathic scoliosis. By integrating measurements ATR with radiographic variables in outcome prediction, the study adopts a multidimensional view of scoliosis management. Both initial and final ATR values demonstrated independent predictive value, underscoring the biomechanical complexity of deformity progression and correction. Furthermore, the findings offer new insights into the impact of curve complexity and predictive modeling of long-term results, thereby advancing the scientific understanding of biomechanical factors in non-operative scoliosis treatment. Our results were in line with the evolving body of evidence on predictors of bracing effectiveness [42,43], which increasingly highlights the need to evaluate not only coronal correction but also rotational behavior and curve-type specificity. Importantly, this study not only confirms previously reported predictors of bracing success but also advances current knowledge. We demonstrate that rotational parameters (ATR) contribute independent prognostic value and, when combined with the six-month out-of-brace Cobb angle, offer a more comprehensive and clinically informative framework for anticipating long-term treatment outcomes.

### 4.4. Previous Research

The importance of baseline factors, such as lower initial Cobb angles and higher Risser scores, in achieving treatment success is well-established. Our findings align with the study by [44], which reported an 83.8% success rate with the Rigo System Chêneau brace and similarly linked success to skeletal maturity and initial curve severity. However, while confirming these baseline relationships, our study emphasizes that the mid-term correction at six months serves as a stronger, more dynamic predictor than baseline factors alone. Both studies confirm the importance of skeletal maturity and initial curve severity, but ours highlights mid-term correction as a stronger predictor than baseline factors alone. The pivotal role of patient compliance in treatment outcome is strongly supported across the literature. Specifically, the findings of [45], which demonstrated success only in compliant patients and highlighted the necessity of early correction (≥40% at 6 months), directly echo our results regarding the critical role of the six-month response. Similarly, the study by [46] using the OMC brace reported significantly better outcomes in patients with adherence > 50%, reinforcing the dose-dependent response observed in our cohort. Furthermore, even in challenging cases, such as bracing large curves (≥40°) as reported by [45], high adherence and early in-brace correction remained key predictors of success. The study by [45] found that brace treatment was only successful in compliant patients (compliance score ≥ 3), achieving an average correction of 10°, compared to no success in non-compliant groups. Early correction (≥40% at 6 months) combined with good compliance led to a mean final correction of 7° (31° to 24°), while poor early correction (<40%) resulted in minimal improvement even with compliance. Both studies agree on the critical role of early treatment response and compliance, and curve-specific outcomes. Study [46] reported a 67.7% success rate with the Osaka Medical College (OMC) brace, with better outcomes in patients adhering >50% (88.2% success vs. 42.8% in ≤50% adherence). Initial Cobb angle (27.3°) remained nearly unchanged at follow-up (28.6°), suggesting stabilization rather than correction. Both studies agree on the importance but differ in correction potential and key prognostic factors. Study [47] reported a 61% success rate in bracing large curves (≥40°), with adherence (17.1 vs. 11.5 h/day) and in-brace correction (≥25% for double curves) as key predictors. Notably, double curves with <25% correction had only a 25% success rate. Regarding the biomechanical potential of bracing, the high in-brace correction rates achieved by advanced designs are notable. The study by [48] on the rigid ARTbrace demonstrated substantial immediate in-brace correction, underscoring the effectiveness of asymmetric rigid design and curve-specific biomechanics. However, a distinction must be made: while such rigid designs achieve high immediate correction, our findings suggest that sustained correction, particularly by six months, offers greater prognostic reliability, supporting a dynamic view of treatment assessment. Our results also complement those of [49], who demonstrated that high-correction Chêneau-style bracing can lead to a significant reduction in vertebral wedging, indicating potential for true structural remodeling, particularly in lumbar curves. Study [46] reported exceptional in-brace correction (72.5% overall) using the rigid ARTbrace, with thoracolumbar/lumbar curves achieving 78.8% correction vs. thoracic curves at 67.6%. This far surpassed conventional braces (e.g., BrAIST 33%). The study emphasized asymmetric rigid design and curve-specific biomechanics as key drivers of correction. However, our findings suggest that sustained correction, especially by six months, offers greater prognostic reliability, supporting a more dynamic view of treatment assessment. The results of our study are consistent with those of [50], who demonstrated that the combination of Chêneau-type bracing and Schroth Best Practice exercises led to a significant improvement in the Cobb angle in adolescents with idiopathic scoliosis, with 88.1% of patients showing curve correction and 83.3% maintaining stability at long-term follow-up. Similarly, our study highlighted the importance of dynamic monitoring, early correction, and individualized treatment, emphasizing the long-term sustainability of conservative management. Both studies underline the feasibility of achieving lasting spinal stabilization with a multidisciplinary, patient-centered approach. Comparably, Ref. [49] reported a 44% reduction in vertebral wedging after high-correction Chêneau-style bracing, indicating potential for true structural remodeling in skeletally immature patients. The effectiveness of our multidisciplinary, patient-centered approach is supported by studies that integrate bracing with specific exercise protocols. For example, Ref. [49] showed that combining Chêneau-type bracing with Schroth Best Practice exercises leads to significant improvement and long-term stability. In a similar vein, Ref. [51] showed that specific exercises (SEAS protocol) could reduce the need for bracing, and our observed dose-dependent response to brace adherence parallels the adherence thresholds noted for exercise efficacy. These observations are consistent with the findings of [52], which reported that the use of the Lyon brace leads to stabilization and correction, attributed to its biomechanical effect on vertebral modeling. Collectively, these studies align with the broader consensus, highlighted by the systematic review [53] and the meta-analysis [54], that bracing is an evidence-based nonoperative treatment effective in preventing curve progression, provided it is managed within a multidisciplinary framework adhering to SOSORT and SRS guidelines. Our results complement these findings: lumbar curves, which are more amenable to derotation, maintained correction more successfully over time, while thoracic curves exhibited regression after initial gains. Finally, study by [51] showed that physiotherapeutic scoliosis-specific exercises (SEAS protocol) could reduce the need for bracing by 1.7-fold with moderate adherence (90 min per week). Although our study did not directly assess specific exercise protocols, the observed dose-dependent response to brace adherence parallels the adherence thresholds noted for exercise efficacy, reinforcing the broader importance of patient compliance. The study by [52] reports that the use of the Lyon brace leads to stabilization and/or correction of thoracic curves in adolescents with idiopathic scoliosis, attributed to its biomechanical effect on vertebral modeling. Their findings also suggest that conservative treatment approaches following both SOSORT and SRS guidelines yield better outcomes than those based on SRS criteria alone, highlighting the importance of these combined standards for future research and methodological consistency in meta-analyses. The systematic review by [53] highlights that bracing is now considered an evidence-based nonoperative treatment for adolescent idiopathic scoliosis, supported by two level A randomized controlled trials. While no specific type of brace has been proven superior, there is consensus on patient management, emphasizing multidisciplinary care, monitoring in-brace correction, and maintaining quality of life. The meta-analysis by [54] concludes that bracing is effective in the treatment of AIS. Rigid full-time braces, rigid night-time braces, and full-time soft braces are all more effective than observation alone in preventing curve progression. Notably, the reported effectiveness of night-time rigid braces is comparable to that of full-time rigid braces, while soft braces appear to be less effective.

### 4.5. Strengths and Limitations

This study possesses several important strengths that enhance the robustness and clinical relevance of its findings. Most notably, it features one of the longest follow-up durations reported in conservative scoliosis treatment research, with an average treatment period exceeding four years. Such an extended observation window allowed for the assessment of not only immediate but also sustained spinal corrections beyond the active bracing phase, providing a rare and valuable perspective on the true durability of conservative interventions. Additionally, the study utilized a rigorous longitudinal design with standardized radiographic measurements at key clinical milestones—initial, four weeks, six months, and final evaluation—capturing the dynamic evolution of spinal curvature over time. The stratification of patients based on curve type and presence of compensatory curves enabled a nuanced analysis of factors influencing treatment success, adding depth and clinical applicability to the results. Moreover, the use of both correlation and multivariate regression analyses strengthened the predictive validity of the findings, particularly the novel demonstration that six-month correction is a more powerful prognostic indicator than initial in-brace correction. Together, these strengths position the study as a significant contribution to advancing evidence-based, individualized approaches in the conservative management of adolescent idiopathic scoliosis.

This study has several limitations. The relatively small sample size (*n* = 50) may limit the statistical power and generalizability of the results to broader populations. Although the multivariate regression model achieved statistical significance and explained a substantial proportion of the variance in treatment outcomes, the number of participants was below the recommended threshold for confidently assessing individual predictor effects. Additionally, the subgroup analyses by curve type, particularly the thoracolumbar subgroup (*n* = 7) should be interpreted with caution due to limited statistical power. Future studies with larger sample sizes are necessary to confirm these findings and enhance generalizability. The observational design, while reflective of real-world clinical practice, introduces potential biases, including selection and recall bias. Additionally, the absence of a comparison group using other brace types or exercise-only protocols restricts the scope of comparison.

The radiological outcomes were assessed only in the frontal plane, as most current clinical protocols and guidelines remain predominantly coronal-based. Using different OOB intervals across time points may introduce limited variability in Cobb angle measurements. However, each interval was intentionally selected to match the biomechanical goals of the corresponding treatment phase, and final outcome interpretation relies primarily on the end-of-treatment radiograph, minimizing the potential impact on overall conclusions. Although the sagittal alignment is increasingly recognized as crucial for spinal balance, its radiographic quantification and standardized follow-up parameters in brace treatment remain insufficiently defined. However, clinical evaluation of the sagittal balance had been ongoing throughout the treatment, making sure that bracing does not negatively affect the sagittal profile inducing hypokyphosis. Additionally, as brace treatment and Schroth physiotherapy were consistently applied together in our clinical setting, their therapeutic effects are inherently interdependent and cannot be analytically separated within the context of this study. Lastly, the single-center design and the relatively specific nature of the patient population (i.e., treatment with the Chêneau modified brace combined with an integrated Schroth-based physiotherapy protocol) may limit the generalizability of these findings to different clinical settings or other brace types. Future multicenter studies involving varied patient cohorts are warranted to validate our results across broader populations.

Furthermore, adherence was assessed only subjectively and limited to the first six months, introducing potential recall and reporting bias. Because such early-phase, self-reported estimates are known to diverge from actual wear-time, compliance was not included in the statistical analyses to avoid misclassification bias. The absence of objective sensor-based monitoring adds some uncertainty regarding the true therapeutic dose; however, the primary endpoint—radiographic correction at the end of treatment—reflects cumulative long-term response and is therefore unlikely to be substantially influenced by this limitation. Future studies incorporating continuous objective monitoring would allow a more precise evaluation of the dose–response relationship.

Although the long-term outcomes were obtained retrospectively, all patients who completed treatment had fully documented radiographic and clinical records, as these assessments are part of our standardized institutional protocol. Therefore, the retrospective nature of this phase did not affect data completeness. The only patients not included in the final analysis were true dropouts—those who discontinued brace treatment—rather than individuals with missing or incomplete documentation.

Attrition represents an inherent limitation of long-term clinical studies. Despite 37.5% of patients discontinuing treatment, sensitivity analyses employing multiple imputation demonstrated that the primary conclusions remained robust. The strong concordance between complete-case and imputed models further supports the six-month Cobb angle as a reliable prognostic indicator, notwithstanding the presence of attrition. Nevertheless, future studies incorporating larger cohorts and objective compliance monitoring are warranted to further mitigate potential attrition bias.

Even though specific reasons for discontinuation of treatment were not documented due to the absence of follow-up information for patients who stopped attending appointments, previous studies suggest that dropout in AIS bracing is most commonly associated with long treatment duration, brace-related discomfort, reduced motivation over time, and psychosocial factors typical of adolescence [31,42,55].

Lastly, the study does not include post-treatment follow-up into adulthood, making it difficult to draw conclusions about the long-term durability of corrections beyond skeletal maturity.

### 4.6. Recommendations

Based on our findings, we propose several clinical and research recommendations. First, clinicians should incorporate six-month out-of-brace Cobb angle measurements as a key decision-making tool when evaluating treatment progression and determining whether to modify the bracing protocol. Second, special attention should be given to patients with thoracic and multiple-curve deformities, as they may require closer monitoring and possibly adjunctive therapies to achieve sustained results. Objective compliance tracking tools should be adopted in future clinical practice to ensure more accurate adherence data. For researchers, future studies should aim for larger, multicenter designs to improve generalizability and should include long-term follow-up into adulthood to assess the durability of conservative treatment. Investigations into the psychological and behavioral aspects of adherence could also help optimize patient-centered bracing protocols.

Future studies would benefit from adopting a curve-specific approach, with separate analyses for major curve types (e.g., thoracic, thoracolumbar, lumbar), as the subgroup sizes in our cohort were relatively small to allow robust stratified comparisons.

Furthermore, integrating emerging 3D measurement technologies into future brace-evaluation protocols may further clarify the relative contributions of coronal, sagittal, and rotational correction to long-term treatment stability [56,57].

## 5. Conclusions

This study demonstrates that conservative brace management can produce significant and sustained radiological and clinical improvements in adolescents with idiopathic scoliosis, particularly when optimal correction is achieved within the first six months of treatment. Patients with single-curve patterns and without compensatory curves exhibited the most favorable outcomes.

Importantly, the six-month out-of-brace Cobb angle proved to be the strongest independent predictor of long-term treatment success, underscoring the importance of dynamic, time-dependent follow-up rather than exclusive reliance on immediate in-brace correction. Incorporating a standardized six-month out-of-brace radiograph into routine clinical protocols may therefore enhance early prognostic assessment and guide individualized treatment adjustments.

Angle of trunk rotation (ATR), both at the start and at the end of therapy, also contributed independently to outcome prediction, highlighting the value of combining clinical and radiographic parameters for comprehensive evaluation. Moreover, the differential responses observed across curve locations suggest that a personalized approach tailoring brace wear schedules, monitoring frequency, and adjunctive physiotherapy to curve type may maximize treatment efficacy.

Despite certain limitations, including the modest sample size and single-center design, the present findings provide valuable evidence supporting a more nuanced, patient-specific model of conservative scoliosis management. Future research should aim to confirm these results in larger, multicenter cohorts and explore the long-term durability of conservative corrections into adulthood. Collectively, these results reinforce the importance of early intervention, individualized treatment planning, and continuous evaluation in optimizing non-surgical outcomes for adolescents with idiopathic scoliosis.

## Figures and Tables

**Table 1:** Analysis of differences according to treatment status (completed vs. dropouts).

Variables	Mean ± SD (Min–Max)		
	Completed (*n* = 50)	Dropouts (*n* = 30)	*p* Value
Age (years)	13.5 ± 1.4 (10–16)	13.2 ± 1.6 (10–15.5)	0.326 ^a^
Sex (female/male)	40/10	25/5	0.941 ^b^
Curve Location	thoracic (*n* = 15)	thoracic (*n* = 8)	0.967 ^b^
	thoracolumbar (*n* = 7)	thoracolumbar (*n* = 4)	
	lumbar (*n* = 28)	lumbar (*n* = 18)	
Risser sign Initial	1.47 ± 1.18 (0–3)	1.25 ± 1.23 (0–3)	0.378 ^c^
Initial Cobb angle	28.7± 7.1 (20–44)	29.2 ± 9.0 (20–45)	0.805 ^a^

^a^ *t*-test, ^b^ Chi-Square, ^c^ Mann–Whitney.

**Table 2:** Study Population Characteristics.

Variables	Mean ± SD (Min–Max)
Age (years)	13.5 ± 1.4 (12.5–16)
Height Initial (cm)	166.83 ± 9.45 (140.5–193.5)
Height Final (cm)	172.96 ± 8.57 (153–203)
Weight Initial (kg)	52.75 ± 12.35 (28–91)
Weight Final (kg)	61.52 ± 12.41 (38–110)
Sex (female/male)	40/10
Curve Location	thoracic (*n* = 15)
	thoracolumbar (*n* = 7)
	lumbar (*n* = 28)
Risser sign Initial	1.47 ± 1.18 (0–3)
Risser sign Final	4.7 ± 0.39 (4–5)
Total duration of treatment (months)	48.29 ± 9.36 (29.77–71.47)

SD—Standard Deviation; Min—minimum; Max—maximum.

**Table 3:** Factors Affecting Scoliosis Correction Outcomes.

Variable	*n*	Correction (Mean ± SD, Min–Max)	*p*
**Number of Curves**			0.014
Single curve scoliosis	11	11.4 ± 6.1° (2–24)	
Multiple curve scoliosis	39	5.3 ± 7.2° (−10–23)	
**Compensatory Curves**			0.023
Present	40	5.5 ± 7.2° (−10–23)	
Absent	10	11.3 ± 6.5° (2–24)	

SD—Standard Deviation; Min—minimum; Max—maximum.

**Table 4:** Cobb Angle Changes Over Treatment Time Points.

Time	Cobb Angle (Mean ± SD)	Comparison	Mean Difference ± SE	CI 95%	η^2^	*p*
Initial	28.7 ± 7.1	Initial vs. 4 weeks	15.96 ± 0.92	(13.44, 18.48)		0.000
		Initial vs. 6 months	7.92 ± 0.81	(5.68, 10.16)		0.000
		Initial vs. Final	6.64 ± 1.04	(3.79, 9.50)	0.87	0.000
4 weeks	12.8 ± 9.1	4 weeks vs. 6 months	−8.04 ± 0.94	(−10.63, −5.45)		0.000
		4 weeks vs. Final	−9.32 ± 1.06	(−12.22, −6.42)		0.000
6 months	20.8 ± 10.5	6 months vs. Final	−1.28 ± 0.95	(−3.89, 1.33)		1
Final	22.1 ± 10.5					
	**ATR (Mean ± SD)**					
Initial	9.00 ± 3.49	Initial vs. Final	3.9 ± 0.56	(2.78, 5.02)	0.5	<0.001
Final	5.1 ± 3.22					

SD—Standard Deviation; SE—Standard Error; CI—Confidence Interval; η^2^—Partial Eta Squared; ATR—Angle of Trunk Rotation.

**Table 5:** Cobb Angle Progression by Spinal Region Over Time.

Region (*n*)	Time Comparison	Cobb Angle (Mean ± SD)	Mean Difference ± SE	CI 95%	η^2^	*p*
Thoracic (15)	Initial vs. 4 weeks	30.47 ± 8.18 → 16.67 ± 9.21	13.80 ± 1.66	(9.238, 18.362)		<0.001
	Initial vs. 6 months	30.47 ± 8.18 → 24.13 ± 11.37	6.33 ± 1.49	(2.231, 10.436)		0.001
	Initial vs. Final	30.47 ± 8.18 → 26.93 ± 10.53	3.53 ± 1.85	(−1.562, 8.629)	0.62	0.373
	4 weeks vs. 6 months	16.67 ± 9.21 → 24.13 ± 11.37	−7.47 ± 1.73	(−12.240, −2.693)		<0.001
	4 weeks vs. Final	16.67 ± 9.21 → 26.93 ± 10.53	−10.27 ± 1.92	(−15.565, −4.969)		<0.001
	6 months vs. Final	24.13 ± 11.37 → 26.93 ± 10.53	−2.80 ± 1.75	(−7.616, 2.016)		0.696
Thoracolumbar (7)	Initial vs. 4 weeks	27.43 ± 8.42 → 12.29 ± 8.12	15.14 ± 2.42	(8.465, 21.820)		<0.001
	Initial vs. 6 months	27.43 ± 8.42 → 18.14 ± 8.86	−9.29 ± 2.18	(−15.291, −3.281)		0.001
	Initial vs. Final	27.43 ± 8.42 → 17.86 ± 10.45	−9.57 ± 2.71	(−17.030, −2.113)	0.48	0.006
	4 weeks vs. 6 months	12.29 ± 8.12 → 18.14 ± 8.86	−5.86 ± 2.54	(−12.845, 1.131)		0.152
	4 weeks vs. Final	12.29 ± 8.12 → 17.86 ± 10.45	−5.57 ± 2.82	(−13.327, 2.184)		0.322
	6 months vs. Final	18.14 ± 8.86 → 17.86 ± 10.45	0.29 ± 2.56	(−6.764, 7.336)		1
Lumbar (28)	Initial vs. 4 weeks	28.11 ± 6.17 → 10.79 ± 8.88	17.32 ± 1.21	(13.983, 20.660)		<0.001
	Initial vs. 6 months	28.11 ± 6.17 → 19.68 ± 10.30	8.43 ± 1.09	(5.426, 11.431)		<0.001
	Initial vs. Final	28.11 ± 6.17 → 20.54 ± 9.93	7.57 ± 1.35	(3.842, 11.301)	0.82	<0.001
	4 weeks vs. 6 months	10.79 ± 8.88 → 19.68 ± 10.30	−8.89 ± 1.27	(−12.387, −5.399)		<0.001
	4 weeks vs. Final	10.79 ± 8.88 → 20.54 ± 9.93	−9.75 ± 1.41	(−13.628, −5.872)		<0.001
	6 months vs. Final	19.68 ± 10.30 → 20.54 ± 9.93	−0.86 ± 1.28	(−4.382, 2.668)		1.000

SD—Standard Deviation; SE—Standard Error; CI—Confidence Interval; η^2^—Partial Eta Squared.

**Table 6:** Longitudinal Cobb Angle Evaluation: Correlation, Odds Ratios, and Regression.

Pearson Correlation					
	Initial	4 Weeks	6 Months	Final	
Initial	1	0.705	0.865	0.717	
4 weeks	0.705	1	0.778	0.72	
6 months	0.865	0.778	1	0.795	
Final	0.717	0.72	0.795	1	
Correlation of Cobb Differences and Later Measurements					
	Initial–4 weeks	Initial–6 months	Initial–Final		
Initial–4 weeks	1	0.412	0.424		
Initial–6 months		1	0.496		
Initial–Final			1		
OR for Final Cobb Angle < 30°					
Variable	OR	SE	*p*-value	95% CI	Pseudo R^2^
Initial	0.759	0.078	<0.001	0.651–0.885	0.36
4 weeks	0.784	0.07	<0.001	0.684–0.899	0.393
6 months	0.726	0.093	0.001	0.605–0.870	0.485
OR for Improvement > 5° Cobb angle					
Change Type	OR	SE	*p*-value	95% CI	Pseudo R^2^
Initial to 4 weeks	1.084	0.048	0.092	0.987–1.192	0.06
Initial to 6 months	1.183	0.065	0.009	1.042–1.344	0.159
OR for Improvement by Correction Rate					
Correction Rate	OR	SE	*p*-value	95% CI	Pseudo R^2^
4 weeks (%)	1.028	0.012	0.022	1.004–1.053	0.113
6 months (%)	1.037	0.015	0.017	1.007–1.069	0.144
Multiple Linear Regression for Final Cobb Angle					
Variable	Crude Coefficient	*p*	Adjusted Coefficient	*p*	VIF
Cobb angle—Initial	1.063	<0.001	0.295	0.193	4.062
Cobb angle—4 weeks	0.832	<0.001	0.235	0.1	2.575
Cobb angle—6 months	0.797	<0.001	0.508	0.004	4.952
ATR—Initial	0.322	0.46	−0.997	<0.001	1.279
ATR—Final	1.547	0.001	0.612	0.004	1.281

SE—Standard Error; OR—Odds Ratio; CI—Confidence Interval; VIF—Variance Inflation Factor; ATR—angle of trunk rotation; Pseudo-R^2^—indicator of model fit.

**Table 7:** ROC Analysis—Predictive Value of Cobb Angle Measurements.

Measurement Time	AUC (Cobb > 30° = Positive)	AUC (Cobb < 30° = Positive)	95% CI	*p*
Initial	0.888	0.112	(0.795, 0.980)	<0.001
4 weeks	0.903	0.097	(0.821, 0.985)	<0.001
6 months	0.944	0.056	(0.882, 1.000)	<0.001

AUC—Area Under the Curve; CI—Confidence Interval.

**Table 8:** Logistic regression sensitivity analysis (complete case vs. multiple imputation).

Predictor	OR (95% CI)—Complete Case	*p* (CC)	OR (95% CI)—Pooled MI	*p* (MI)
Initial Cobb angle	0.759 (0.651–0.885)	<0.001	0.799 (0.670–0.954)	0.017
4-week Cobb angle	0.784 (0.684–0.889)	<0.001	0.827 (0.739–0.925)	0.002
6-month Cobb angle	0.726 (0.605–0.870)	0.001	0.742 (0.607–0.905)	0.006

OR—Odds Ratio; CI—Confidence Interval; CC—Complete Case Analysis; MI—Multiple Imputation Analysis.

## Data Availability

The original contributions presented in this study are included in the article. Further inquiries can be directed to the corresponding authors.

## Abbreviations

The following abbreviations are used in this manuscript:

3D	three-dimensional
AIS	adolescent idiopathic scoliosis
AP	anteroposterior
ATR	angle of trunk rotation
BSPTS	Barcelona Scoliosis Physical Therapy School
CAD/CAM	computer-aided design/computer-aided manufacturing
CMB	Chêneau modified brace
CI	confidence interval
FAMA	Functionally Aware Motoric Activity
OOB	Out of brace
OMC	Osaka Medical College (brace)
OR	odds ratio
PRM	Physical and Rehabilitation Medicine
PSSE	physiotherapeutic scoliosis-specific exercises
SEAS	Scientific Exercises Approach to Scoliosis
SRS	Scoliosis Research Society
SOSORT	Society on Scoliosis Orthopaedic and Rehabilitation Treatment
SPSS	Statistical Package for the Social Sciences
XR	X-ray
BrAIST	Bracing in Adolescent Idiopathic Scoliosis Trial
